# Status of Intestinal Parasitic Infections among Primary School Children in Rivers State, Nigeria

**DOI:** 10.1155/2015/937096

**Published:** 2015-10-27

**Authors:** A. E. Abah, F. O. I. Arene

**Affiliations:** Department of Animal and Environmental Biology, Faculty of Science, University of Port Harcourt, PMB 5323, Port Harcourt 500001, Rivers State, Nigeria

## Abstract

Status of intestinal parasitic infections among primary school children in Rivers State, Nigeria, was investigated between January and December 2011. A total of 3,826 stool samples were collected from school children (1,828 males and 1998 females) in 36 primary schools from 13 local government areas of Rivers State. The samples were analyzed using wet saline/iodine and formol ether concentration methods. Of the 3,826 stool samples examined, 1059 (27.66%) were positive for different intestinal parasites, namely, *Ascaris lumbricoides* (51.78%), hookworm sp. (25.0%), *Trichuris trichiura* (15.18%), *Strongyloides stercoralis* (7.14%), *Taenia* sp. (0.89%), and *Enterobius vermicularis* (0.01%). The prevalence of the infection was generally higher in males (57.60%) than females (42.40%). The differences were not statistically significant (*P* > 0.05). Among these intestinal parasites, *Ascaris lumbricoides*, hookworm sp., and *Trichuris trichiura* were found in all the 13 local government areas studied while *Strongyloides stercoralis* was found in 12, *Taenia* sp. in five, and *Enterobius vermicularis* in only one community in Ahoada Local Government Area. The overall infection rate remains high and would require coordinated deworming of the school children within the state.

## 1. Introduction

Few reports exist on intestinal parasitic infections among school children in Rivers State, Nigeria [1–3]. Intestinal parasitic infections have been reported to have high prevalence among children in Nigeria because of their vulnerability [4–9]. Intestinal parasitic infection is known to cause deleterious effects especially in children some of which include trauma, nutrition-robbing and poisoning [10], change in resistance, and immune impotence [11]. These parasites frequently encountered are* Ascaris lumbricoides*,* Trichuris trichiura*,* Strongyloides stercoralis*, and hookworm [12].

Abah and Arene [1] in their work on intestinal helminthiasis among primary school children in Akpor area of Port Harcourt, Rivers State, recorded 42.7% prevalence with hookworm 16.0%,* Ascaris lumbricoides* 15.4%,* Trichuris trichiura* 8.0%,* Strongyloides stercoralis* 3.0%, and* Taenia saginata* 1.7%. Ezenwaka et al. [13] reported 18.5% prevalence among children in Ogbaru Local Government Area of Anambra State with* A. lumbricoides* 9.5%, hookworm 7.5%,* Trichuris trichiura* 1.5%,* E. vermicularis* 1%, and* Taenia* species 1% while Ezeagwuna et al. [14] reported 47% prevalence among school children in Umuukwu, Aram, in Anambra State in their work, the unartificial impact of intestinal helminthiasis among the school children in the area. Ogonaka et al. [15] in “Prevalence of Soil Transmitted Helminthes among Primary School Pupils in Owerri West Local Government Area in Imo State, Nigeria” recorded 35.43%. Opara et al. [16] reported an overall prevalence of 58% among children in day-care centers in Owerri municipality, Imo State, Nigeria. However, this high prevalence is not peculiar to Nigeria but found wherever sanitary and environmental conditions are low.

Tappe et al. [17] reported 42.5% in Urmia region of Iran; Gamboa et al. [18] recorded 73.0%, 54.4%, and 35.2%, respectively, in different zones in La Plata, Argentina. Abu Mourad [19] in his work “Palestinian Refugee Conditions Associated with Intestinal Parasites and Diarrhoea: Nuseirat Refugee Camp as a Case Study” reported 24.1% among children aged 1–4 years. Nkengazong [20] reported 42.4% among children in Kotto Barombi and marimba II villages in Southwest Cameroon. Saathoff et al. [21] reported 83.2% for hookworms' infections in rural Kwazulu-Natal, South Africa.

Since the World Health Organization declared the Sanitation Decade (1981–1990), the Nigerian Government has approached the issue from various perspectives and even declared the monthly National Sanitation as a means of improving the sanitary condition in towns and villages in the country. The Sanitation policy of the government has been steady. In Rivers State, it has been fought in various phases including deworming of primary school's pupils. Responsible government and educated people may understand the implication of poor sanitation. Genuine effort may have been made by the government towards the improvement of standard of environmental sanitation but to what extent the efforts have impacted the people* vis*-à-*vis* the occurrences of the intestinal parasitic infections needs to be ascertained, hence the interest of the present study. This study was aimed at ascertaining the current status of the disease in the children after those interventions.

## 2. Materials and Methods

Permission for the study was sought and obtained from the Rivers State Ministry of Health as well as the heads of the primary schools. The class teachers helped in the distribution of sample bottles to the pupils serially according to their names in the class register. The pupils were instructed on how to collect the samples (i.e., put a small portion of early morning stool sample in the bottle using the spatula attached to the sample bottle and bring it to school). The samples were collected and processed on the same day. Samples that were not to be processed on the same day were preserved in the refrigerator.

Visual examination of the stool sample was carried out, noting the appearance, the colour, the consistency (whether formed, semiformed, unformed, or watery), and the presence or absence of blood, mucus, and pus. Any abnormalities were recorded.

A drop of physiological saline was placed on a clean grease-free slide. Using an applicator stick, a little quantity of properly mixed stool sample was collected and emulsified on the drop of the saline. The preparation was covered with a coverslip and examined with light microscopy at 100x and finally with 400x magnifications.

All the samples were concentrated using formol ether concentration technique. One milliliter of a well-mixed stool sample was put in a tube containing 4 mL of 10% formalin. Three milliliters of the 10% formalin was again added and mixed by shaking. The suspension was sieved using a coffee strainer into a centrifuge tube. Three milliliters of diethyl ether was added and stoppered. It was then shaken vigorously for 1 min. The stopper was removed and the suspension centrifuged for 1 min at 400 rpm. The entire column of the fluid below the faecal debris and ether was carefully removed using a Pasteur pipette and transferred into another centrifuge tube. Ten percent formalin was added to the transferred suspension to make up to 10 mL. It was then centrifuged at 1000 rpm for 10 mins. The supernatant was decanted and the bottom of the tube tapped to resuspend the deposit. The deposit was examined by light microscopy at 100x and 400x magnifications for the presence of ova or cyst of parasites.

## 3. Results

**Figure 1:**
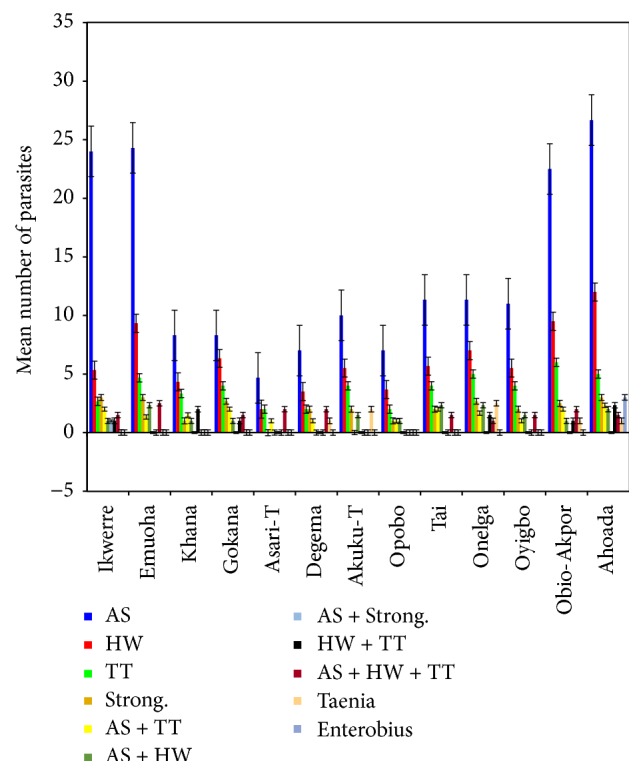
Mean number of types of parasites found in various local government areas of Rivers State, Nigeria.

Of the 3,826 children examined for intestinal parasitic infections, 1059 (27.66%) were infected (Table 1). Parasites identified in the study were* Ascaris lumbricoides* 51.78%, hookworm 25%,* Trichuris trichiura* 15.18%,* Strongyloides stercoralis* 7.14%,* Taenia* sp. 0.89%, and* Enterobius vermicularis* 0.01% (Figure 1).

**Figure 2:**
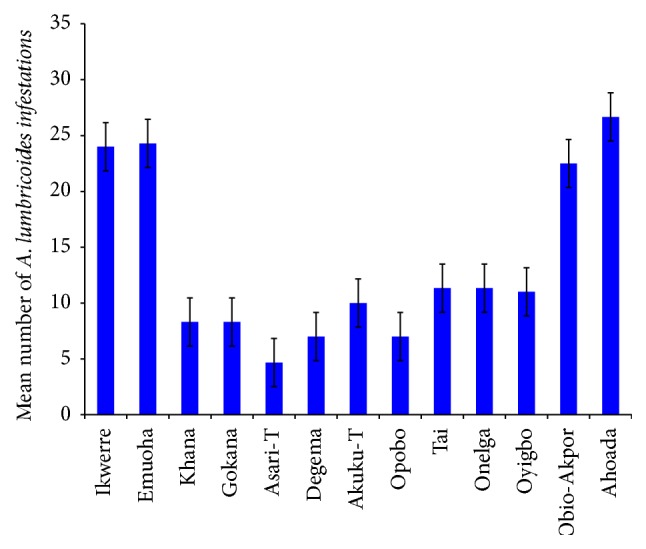
Mean number of* Ascaris lumbricoides* infestations in various local government areas of Rivers State, Nigeria.

**Figure 3:**
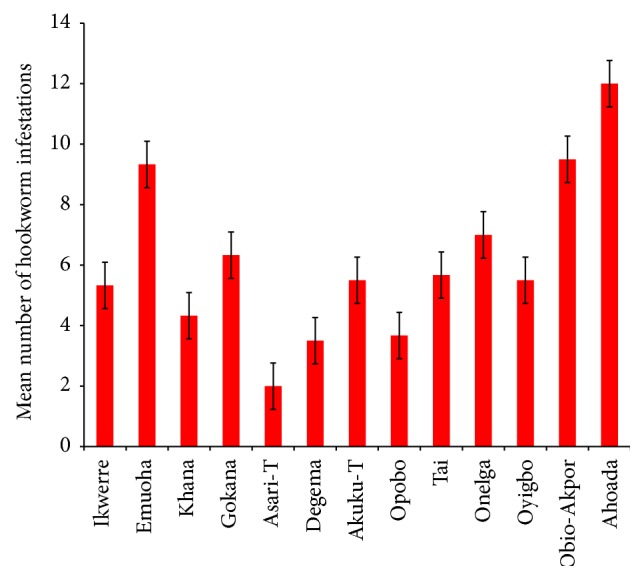
Mean number of hookworm infestations in various local government areas of Rivers State, Nigeria.

**Figure 4:**
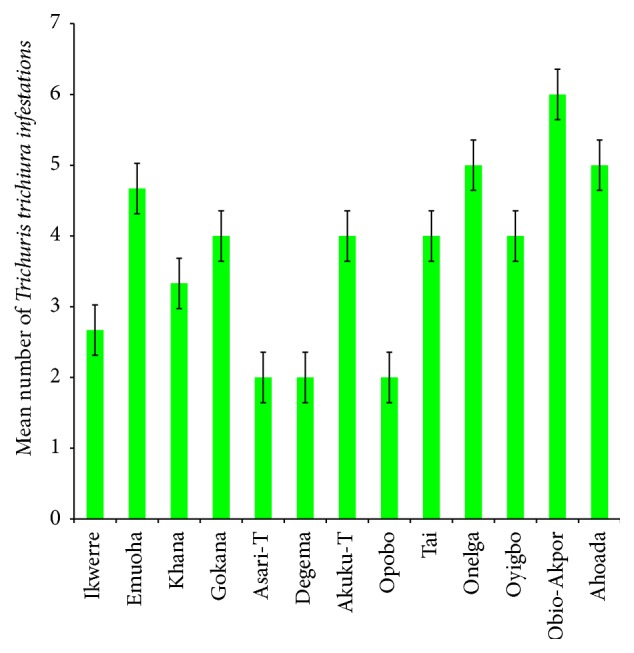
Mean number of* Trichuris trichiura* infestations in various local government areas of Rivers State, Nigeria.

**Figure 5:**
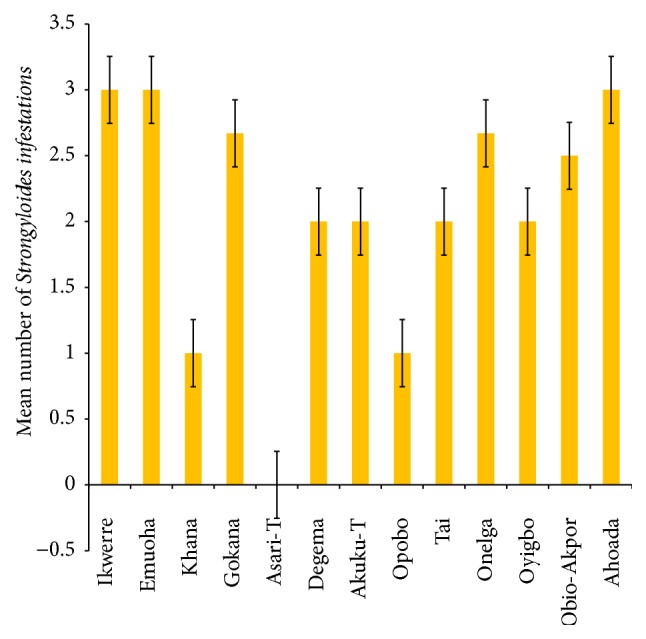
Mean number of* Strongyloides stercoralis* infestations in various local government areas of Rivers State, Nigeria.

**Figure 6:**
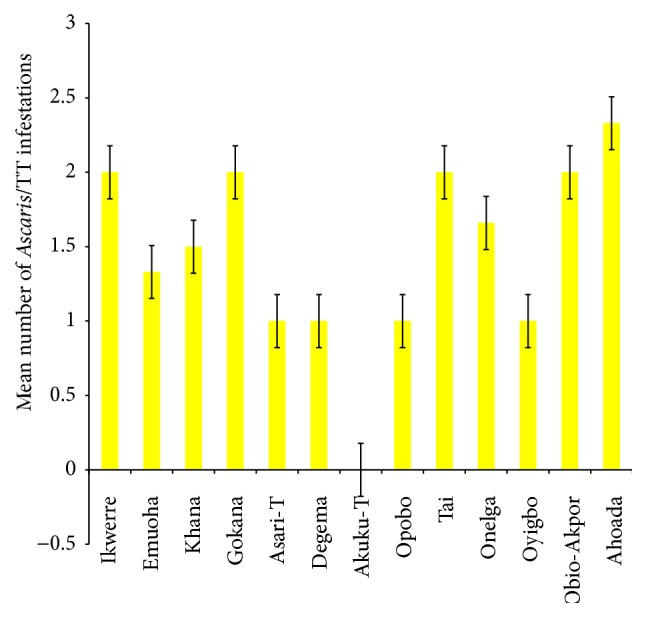
Mean number of* Ascaris lumbricoides* and* Trichuris trichiura* infestations found in the various local government areas of Rivers State, Nigeria.

**Figure 7:**
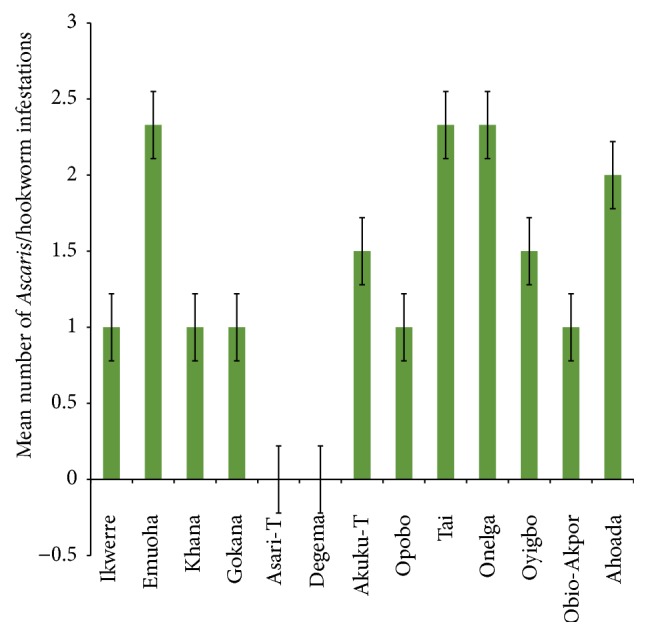
Mean number of* Ascaris lumbricoides* and hookworm infestations in various local government areas of Rivers State, Nigeria.

**Figure 8:**
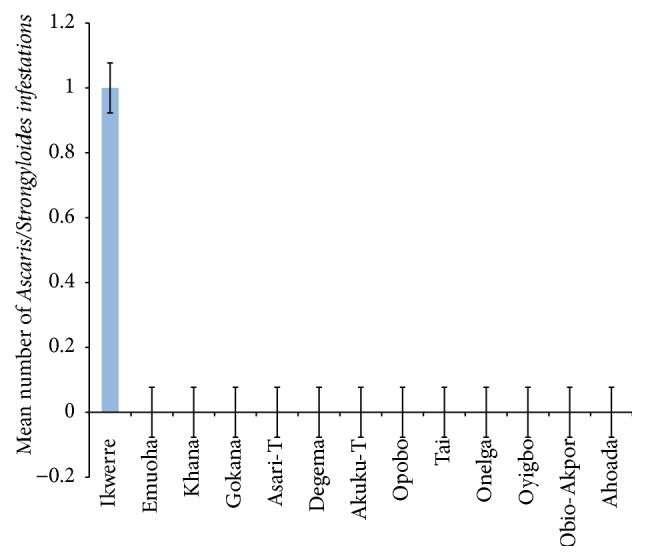
Mean number of* Ascaris lumbricoides* and* Strongyloides stercoralis* infestations in various local government areas of Rivers State, Nigeria.

**Figure 9:**
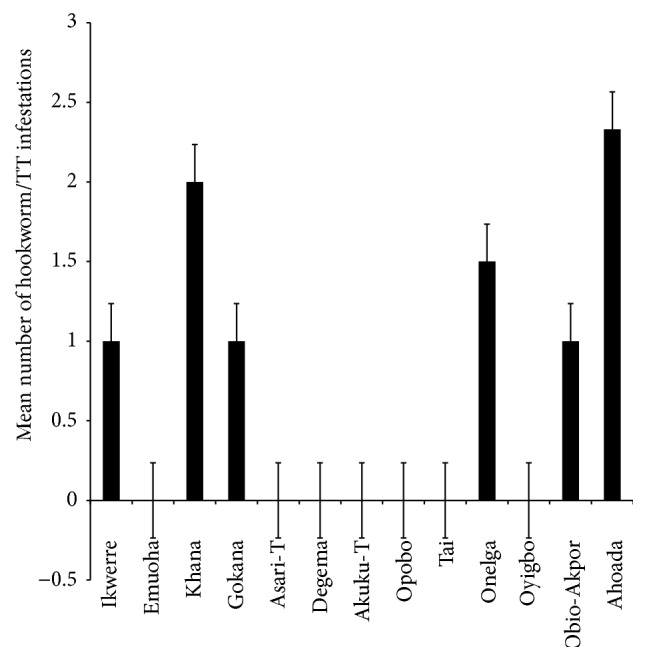
Mean number of hookworm and* Trichuris trichiura* infestations in various local government areas of Rivers State, Nigeria.

**Figure 10:**
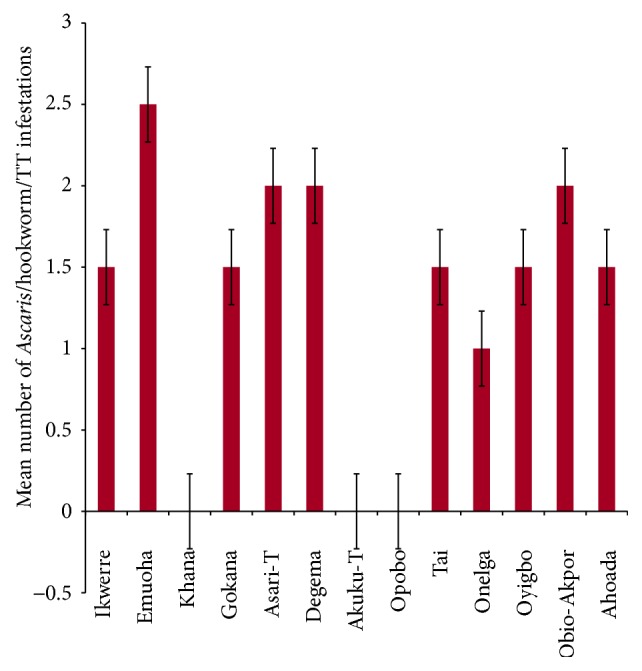
Mean number of* Ascaris lumbricoides,* hookworm, and* Trichuris* infestations in various local government areas of Rivers State, Nigeria.

**Figure 11:**
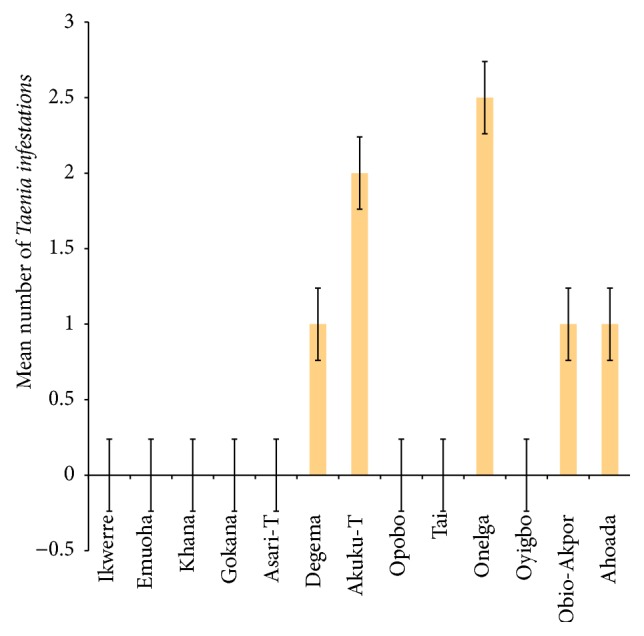
Mean number of* Taenia* sp. infestations in various local government areas of Rivers State, Nigeria.

**Figure 12:**
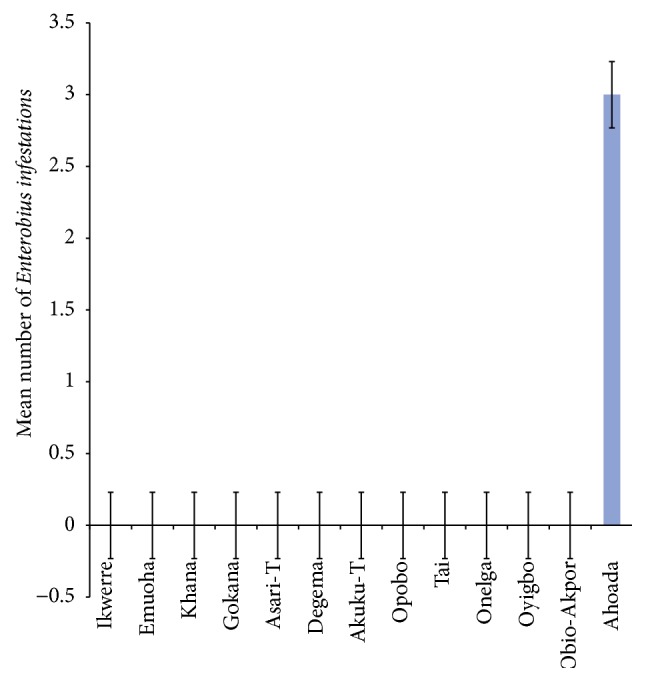
Mean number of* Enterobius vermicularis* infestations in various local government areas of Rivers State, Nigeria.

The mean number of* Ascaris lumbricoides* infections across the local government area is shown in Figure 2, while Figures 3, 4, and 5 show the mean number of hookworm,* Trichuris trichiura*, and* Strongyloides stercoralis* infestations, respectively. Multiple infections were observed and the combinations observed include* Ascaris lumbricoides/Trichuris trichiura* (Figure 6) and* Ascaris lumbricoides*/hookworm (Figure 7), while Figures 8, 9, and 10 are multiple infections of* Ascaris lumbricoides/Strongyloides stercoralis*, hookworm/*Trichuris trichiura*, and* Ascaris lumbricoides*/hookworm/*Trichuris trichiura*, respectively. Figures 11 and 12 show the mean number of* Taenia* sp. and* Enterobius vermicularis* infections in the study area. Out of these intestinal parasites,* Ascaris lumbricoides*, hookworm, and* Trichuris trichiura* were found in all the local government areas studied.* Strongyloides stercoralis, Taenia* sp., and* Enterobius vermicularis* were not found in all the sampled local government areas.* E. vermicularis* was found only in one community.

## 4. Discussion

Microscopic examination of stool samples by direct saline wet preparation/iodine wet preparation and formol ether concentration methods for 3,826 school children in Rivers State, Nigeria, from the thirteen local government areas revealed an overall prevalence of 1059 (27.66%). This result confirms the high endemicity of intestinal parasitic infections which has been reported in Rivers State [1–3]. This endemicity is not restricted to Rivers State as intestinal parasitic infections have been described to be among the most common infections worldwide [22–24] and particularly a tropical disease where a combination of hot, humid climate, poor sanitation and personal hygiene, and ignorance still exists [25].

The prevalence rate of 27.66% shows a reduction when compared with previous works by Awi-Waadu [2] who reported 84.6%, Abah and Arene [1] 42.7%, and Odu et al. [3] 30.7%. This may not be unconnected with recent effort by the Rivers State Government to reduce infant mortality and improve environmental sanitation and personal hygiene via construction of modern classroom in all communities of the state and the schools deworming programme of the wife of the governor and the feeding of school children. All these listed steps and the subsequent reduction are very important because intestinal parasitic infections cause malnutrition, growth deficits, poor educational achievement, and poor cognitive skill [26–28]. Even though there is a reduction, the prevalence of 27.66% is still considered high. This is not surprising because many researchers have reported high prevalence of intestinal parasitic infections in Nigeria and among children due to their vulnerability [4, 6, 7, 29].

Parasites identified in this study were* Ascaris lumbricoides*, hookworm,* Trichuris trichiura*,* Strongyloides stercoralis*,* Taenia* sp., and* Enterobius vermicularis*. Among these parasites,* Ascaris lumbricoides*, hookworm sp., and* Trichuris trichiura* were encountered in all the local government areas sampled. These three parasites are the most common intestinal parasites in the tropics. This observation agrees with some of the previous findings [1–3, 12, 30–33]. Poor environmental hygiene and socioeconomic conditions are among the factors that promote the survival and transmission of these parasites.* Taenia* sp. was found in five out of the thirteen local government areas sampled while* Enterobius vermicularis* was found in only a rural community in Ahoada Local Government Area. The low prevalence rate of* Enterobius vermicularis* may not be unconnected with the examination procedure adopted for this study as swab, clear adhesive tape, or other adaptive methods of the anal region are recommended for the recovery of* Enterobius* ova. It is only occasionally that eggs of* E. vermicularis* can be found in faeces [34]. Previous works suggest that the highest number of eggs can usually be recovered in the morning soon after waking up and before bathing.

## Figures and Tables

**Table 1:** Population examined in the study area.

Total examined	Total positive	Total male	Total female	Total male +ve	Total female +ve	Total negative

3826	1059	1828	1998	610	449	2767
